# Simple and effective exercise design for assessing *in vivo* mitochondrial function in clinical applications using ^31^P magnetic resonance spectroscopy

**DOI:** 10.1038/srep19057

**Published:** 2016-01-11

**Authors:** Alison Sleigh, Victoria Lupson, Ajay Thankamony, David B. Dunger, David B. Savage, T. Adrian Carpenter, Graham J. Kemp

**Affiliations:** 1 Wolfson Brain Imaging Centre, University of Cambridge School of Clinical Medicine, Cambridge Biomedical Campus, Cambridge, CB2 0QQ, UK; 2 National Institute for Health Research/Wellcome Trust Clinical Research Facility, Cambridge University Hospitals NHS Foundation Trust, Cambridge Biomedical Campus, Cambridge, CB2 0QQ, UK; 3 Department of Paediatrics, University of Cambridge, Cambridge Biomedical Campus, Cambridge, CB2 0QQ, UK; 4 University of Cambridge Metabolic Research Laboratories, Wellcome Trust-Medical Research Council Institute of Metabolic Science, Cambridge, CB2 0QQ, UK; 5 Magnetic Resonance & Image Analysis Research Centre, Department of Musculoskeletal Biology and MRC – Arthritis Research UK Centre for Integrated research into Musculoskeletal Ageing, University of Liverpool, Liverpool, L69 3GE, UK

## Abstract

The growing recognition of diseases associated with dysfunction of mitochondria poses an urgent need for simple measures of mitochondrial function. Assessment of the kinetics of replenishment of the phosphocreatine pool after exercise using ^31^P magnetic resonance spectroscopy can provide an *in vivo* measure of mitochondrial function; however, the wider application of this technique appears limited by complex or expensive MR-compatible exercise equipment and protocols not easily tolerated by frail participants or those with reduced mental capacity. Here we describe a novel in-scanner exercise method which is patient-focused, inexpensive, remarkably simple and highly portable. The device exploits an MR-compatible high-density material (BaSO_4_) to form a weight which is attached directly to the ankle, and a one-minute dynamic knee extension protocol produced highly reproducible measurements of post-exercise PCr recovery kinetics in both healthy subjects and patients. As sophisticated exercise equipment is unnecessary for this measurement, our extremely simple design provides an effective and easy-to-implement apparatus that is readily translatable across sites. Its design, being tailored to the needs of the patient, makes it particularly well suited to clinical applications, and we argue the potential of this method for investigating *in vivo* mitochondrial function in new cohorts of growing clinical interest.

Mitochondrial dysfunction is increasingly recognised as important in a variety of diseases including neurodegenerative, cardiovascular and metabolic disorders^1^,^2^,^3^,^4^. ^31^P magnetic resonance spectroscopy (^31^P-MRS) has long been used to assess skeletal muscle mitochondrial function *in vivo* by measuring the kinetics of replenishment of the phosphocreatine (PCr) pool after exercise, which relates directly to oxidative ATP synthesis (reviewed extensively in^5^). The non-invasive nature of this technique permits multiple measurements in longitudinal intervention trials and arguably offers a more representative assessment of muscle mitochondrial function as it reflects the effects of an integrated cardiorespiratory/vascular system and permits a larger sample size than muscle biopsy. The growing recognition of the contribution of mitochondrion-associated diseases to the public health burden makes the use of this technique in clinical trials more important than ever.

A limitation to the wider application of the technique remains the need for MR-compatible exercise equipment suitable for use in clinical contexts. Commercial MR-compatible ergometers are increasingly available^6^,^7^,^8^, but these can be expensive and there is a long tradition of in-house construction of exercise equipment^9^,^10^,^11^,^12^,^13^,^14^,^15^,^16^,^17^,^18^,^19^,^20^,^21^,^22^,^23^,^24^. Exercise may be static (isometric) or dynamic (isotonic); isometric exercise is conveniently scaled to maximum voluntary contraction *in situ*^10^,^11^,^14^,^16^,^17^,^18^,^24^, but adjusting exercise intensity by interpreting force feedback^10^,^13^,^14^,^16^,^18^,^23^ can be challenging. Calibrated dynamic exercise typically requires complex resistance equipment^7^,^11^,^16^,^17^ or pulley systems to transfer the workload outside the bore^15^,^19^,^20^,^22^, and in all systems participant posture is an important comfort consideration.

PCr recovery kinetics following exercise in which cell pH does not change much is well-known to be first-order, characterized by an exponential rate constant (k_PCr_) that is independent of the degree of PCr depletion, and which is directly proportional to what may be physiologically regarded as functional mitochondrial capacity, generally insensitive to the details of the exercise perturbation^5^. Thus to assess muscle mitochondrial function needs only a way of producing moderate PCr depletion, which can be achieved by simple exercise methods rather than requiring sophisticated equipment. Here we exploit this and set out to design a simple, patient-friendly MR exercise method that is inexpensive and permits measurements of *in vivo* mitochondrial function easily in a clinical context where patients may have limited exercise tolerance and/or reduced mental capacity.

## Methods

### Participants

26 (24 M, 2 F) participants with mean ± SEM age 34.9 ± 1.7 y undertook ^31^P-MRS measurements on a Siemens MAGNETOM 3 T scanner. The participants consisted of one 30 y female control subject with a BMI of 21.3 kg/m^2^ who undertook 2.5 minutes of exercise in a Siemens 3 T Trio scanner, one female 40 y lipodystrophic patient (BMI 20.3 kg/m^2^) who performed the optimised exercise protocol in a Siemens 3 T Verio scanner, and 24 healthy non obese Caucasian male volunteers who underwent the optimised exercise protocol on two consecutive days in a Siemens 3 T Verio scanner.

Each participant provided written informed consent and all studies were conducted in accordance with the Declaration of Helsinki. Ethical approval was granted by Cambridgeshire 2 Research Ethics Committee, United Kingdom (for the control subject dataset shown), by the National Health Service Research Ethics Committee, United Kingdom (patient dataset shown), and by Cambridge Local Research Ethics Committee, United Kingdom (the 24 individuals who comprise the reproducibility statistics).

### Workload methodology

**Figure 1:**
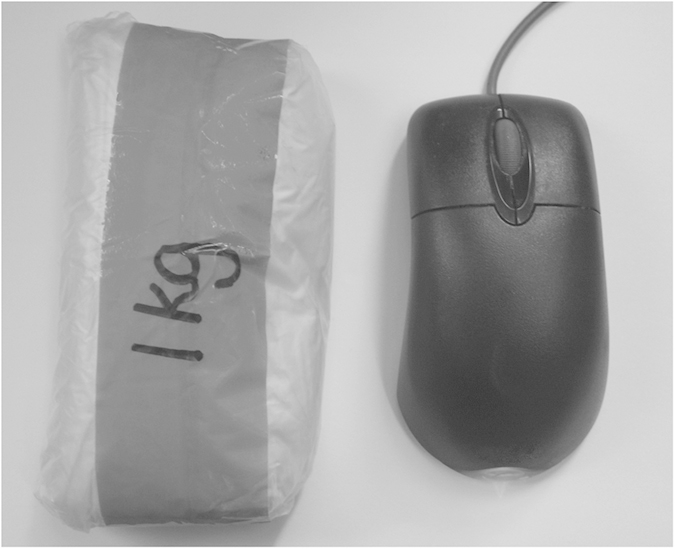
Innovative MR-compatible high density material barium sulfate. Barium sulfate (BaSO_4_) of mass 1 kg placed within a sealed freezer bag (shown next to a computer mouse for context).

This design exploits the high density of the MR-compatible material barium sulfate (BaSO_4_), whose specific gravity is 4.5 (cf. 1.0 for water and 1.5 for dry sand^25^). This permits enough mass (workload) to be placed within the scanner bore and circumvents the need for complex and/or expensive resistance equipment or pulley systems. Barium sulfate is relatively inexpensive and although pure BaSO_4_ is MR-safe, any purchased BaSO_4_ that might contain magnetically susceptible impurities should be tested. To permit an individual-specific workload, the barium sulfate was placed in sealable freezer bags (Fig. 1) following relevant safety data sheet advice, to form bags of differing weights (e.g. 1.00, 0.50, 0.20, 0.15, 0.10, 0.05 kg).

### Exercise design

**Figure 2:**
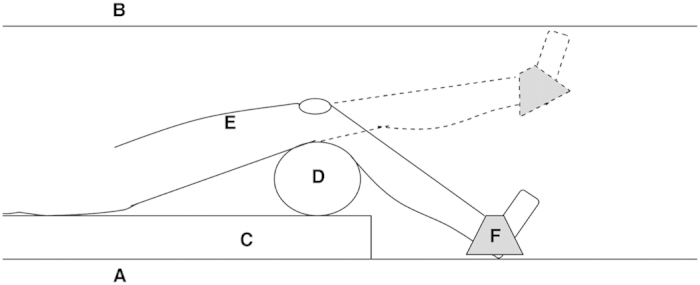
Schematic representation of the exercise design. Sagittal cross sectional profile through the scanner bore showing the exercise design *in situ*. The patient performs knee extension from the table base (**A**) to full knee extension (**B**), with a typical 45° angle of rotation resulting (Siemens MAGNETOM 3 T Verio). The body of the patient lies on the vendor’s standard foam padding (**C**), displaced left such that their right leg is as near to isocentre as feasible. The posterior aspect of their knees rest over a firm cylindrical foam (**D**) of diameter 14.5 cm (standard Siemens equipment), with their ankle resting in the resulting recess in order to maximise exercise rotation. The coil is attached to the participant’s quadriceps (**E**) over the muscle group of interest, and the MR-compatible weight and holder placed over the ankle (**F**).

**Figure 3:**
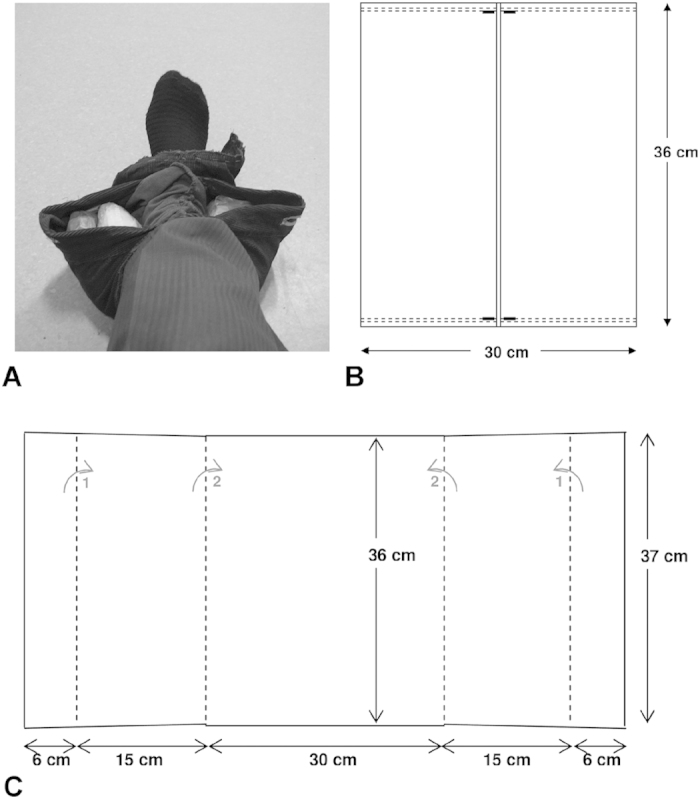
The dual-pocket ankle weight holder. The dual-pocket ankle weight holder filled with 3.0 kg (1.5 kg either side), placed over the ankle (**A**). Schematic representation illustrating dimensions of the built ankle weight holder when laid flat (**B**), and material prior to assembly (**C**). The dashed lines in (**B**) represent the location of strong machine stitching, and in (**C**) identify the material fold lines such that fold 1 (grey arrow), then fold 2 (grey arrow) are performed prior to stitching as denoted in (**B**). Fold 1 aids in ensuring the barium sulfate parcels remain in the holder with heavy workloads.

The participants were positioned in the comfortable feet-first supine position with their knees placed over a 14.5 cm diameter cylindrical foam and their feet placed in the resulting recess, as shown in Fig. 2. This configuration minimizes axial surface coil orientations and provides a wide angle of rotation for knee extension exercise. The appropriate mass (see Section ‘Ankle weight calculation’) was placed over the participant’s right ankle using a custom made dual-pocket ‘bag’ made from non-stretchable cord denim (Fig. 3A), with equal weights of BaSO_4_ either side of the ankle. Figure 3B,C shows dimensions of the holder which can hold sufficient mass (up to ~9 kg), without the bag wrapping under the ankle. We found that the ankle weight did not move significantly relative to the leg during exercise.

### Exercise MRS protocol

**Figure 4:**
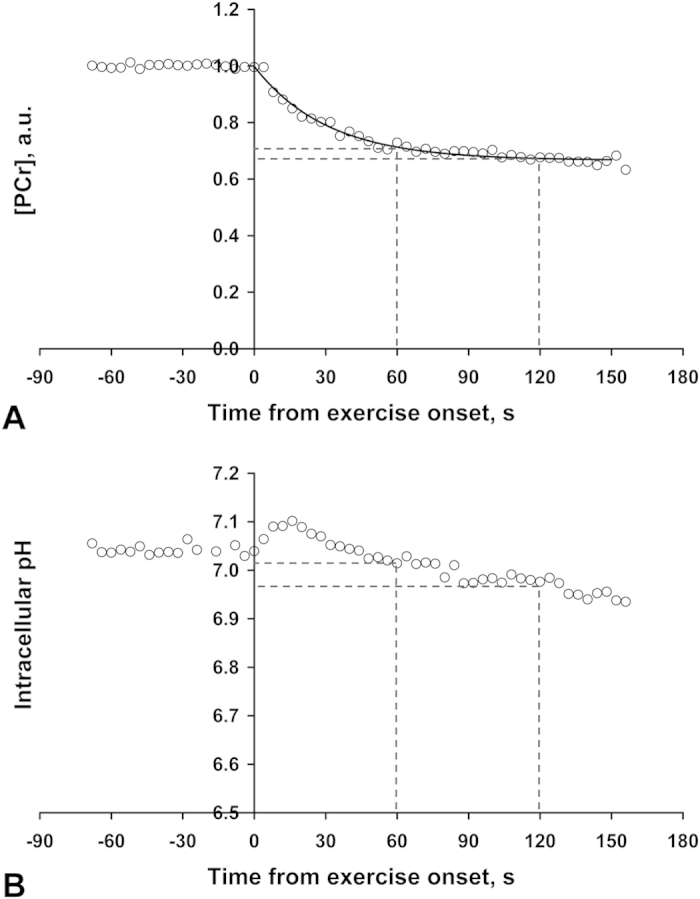
Phosphocreatine (PCr) and pH timecourse prior to and during exercise in an adult control. White circles represent [PCr] (**A**) and intracellular pH (**B**), as determined from individual spectra of ^31^P-MRS data with a TR of 4s. Data acquired on a Siemens MAGNETOM 3T Trio (Erlangen, Germany) in a healthy adult volunteer performing exercise as outlined in Methods: Exercise MRS protocol, with a total ankle weight of 2.7 kg. The solid line in (**A**) represents a mono-exponential fit to the PCr exercise data. Dashed lines aid visualization as to the fractional PCr depletion (**A**) and intracellular pH (**B**), after both 1 and 2 minutes of exercise.

The exercise consisted of knee extensions of the right leg from the scanner table base to full knee extension, with the appropriate weight placed over the right ankle. This was performed at 0.5 Hz and gated such that, with subject compliance, the MR acquisition always occurred with the leg in the ‘down’ position. This was achieved using pre-recorded headphone instructions of up/down commands and warning of exercise onset and cessation. All participants were shown a training video prior to the scan to ensure they were familiar with the exercise protocol and undertook a brief practice before full entry to the scanner bore. One healthy female performed 2.5 minutes of this exercise and dynamic measurements of [PCr] and pH recorded (Fig. 4). From this an optimised exercise protocol was developed.

### Ankle weight calculation

Volunteers’ maximal voluntary contraction (MVC) was determined the previous day using a dynamometer chair set to knee extension from –45° to 0°, where 0° is knee extension without hyperextension (i.e. similar angles of exercise as in the scanner). Participants undertook an isokinetic protocol consisting of three knee extensions with gentle encouragement, and peak torque from their best repetition was recorded as their MVC. Standardisation of PCr depletion between participants was achieved by altering the ankle weight to a fraction of their MVC such that m = 0.131 MVC/(g.L), where m is the ankle weight in kg, MVC the torque in Nm, g is 9.81 ms^−2^, and L is the leg length in meters defined as the lateral femoral condyle to malleolus distance. In the study of 24 healthy non-obese male volunteers, who had height (mean ± SEM) 180.5 ± 1.5 cm, leg length was not measured and L was assumed to be 42 cm, thereby providing m = 0.0318 MVC. The target PCr depletion was 20–25% of basal [PCr] and more than 40% depletion was undesired.

### ^31^P-MRS acquisition and analysis parameters

A 9 cm diameter ^31^P (with butterfly-design ^1^H) transmit-receive flex coil was used for all participants. Data acquisition and analysis methods were as in^1^,^26^, but here data have been averaged to an effective time resolution of 8 s prior to PCr recovery fitting with a monoexponential function (where the mean end-recovery PCr was calculated from the post exercise 8 s averaged time points 155–219 s inclusive). The intracellular pH was determined from the chemical shift of inorganic phosphate relative to PCr^27^. The rate constant of post-exercise PCr resynthesis, k_PCr_, was found using a two parameter monoexponential fit as previously described^26^, and is taken as a measure of muscle mitochondrial function^5^.

### Reproducibility of the PCr recovery rate constant

The level of agreement in k_PCr_ repeatability (within a test session) and reproducibility (between test sessions) were assessed from a study of 24 individuals who underwent the optimised exercise protocol on two consecutive days as part of a fasting study (at 8 and 28 h of fasting)^28^. Bland-Altman analysis was performed and the mean difference (bias) between the two measures was calculated then tested against zero using a one sample t-test. Individuals’ CV of repeatability was assessed as the SD/mean of the two k_PCr_ values obtained at their Day-1 scan. The individuals’ mean k_PCr_ for both Day-1 and Day-2 were then used for an estimate of CV of reproducibility with the fasting intervention of the study (which was found to have no significant effect overall on PCr recovery); this is therefore an upper limit on CV without any intervention.

### Statistics

Statistical analysis was performed in IBM SPSS Statistics 21 (IBM Inc., Armonk, NY, USA), with significance set at p < 0.05. Quantitative data are presented as mean ± SEM, unless otherwise stated.

## Results

### Exercise design

All participants found the exercise comfortable and completed the exercise protocol with no reported negative feedback. The Siemens standard 14.5 cm diameter foam cylinder placed under the volunteers’ knee (Fig. 2) appeared to be appropriate for patient comfort whilst having enough solidity to minimise foam compression at the highest workloads.

**Figure 5:**
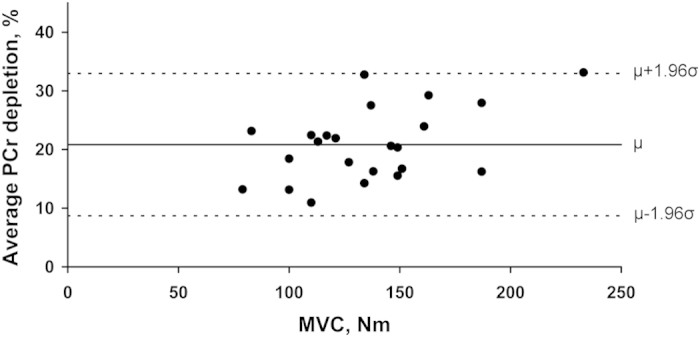
Percentage phosphocreatine (PCr) depletion after one minute of in-scanner exercise against participants’ previously determined maximal voluntary contraction (MVC) in 23 healthy males. Black circles represent individuals’ average PCr depletion on Day-1 (relative to basal levels) after performing one minute of exercise (optimised exercise protocol) with ankle weight as determined in Section ‘Methods: Ankle weight calculation’, against their MVC as assessed from the dynamometer chair. The solid line represents the mean PCr depletion (μ), and dashed lines the mean PCr depletion ± 1.96 SD.

**Figure 6:**
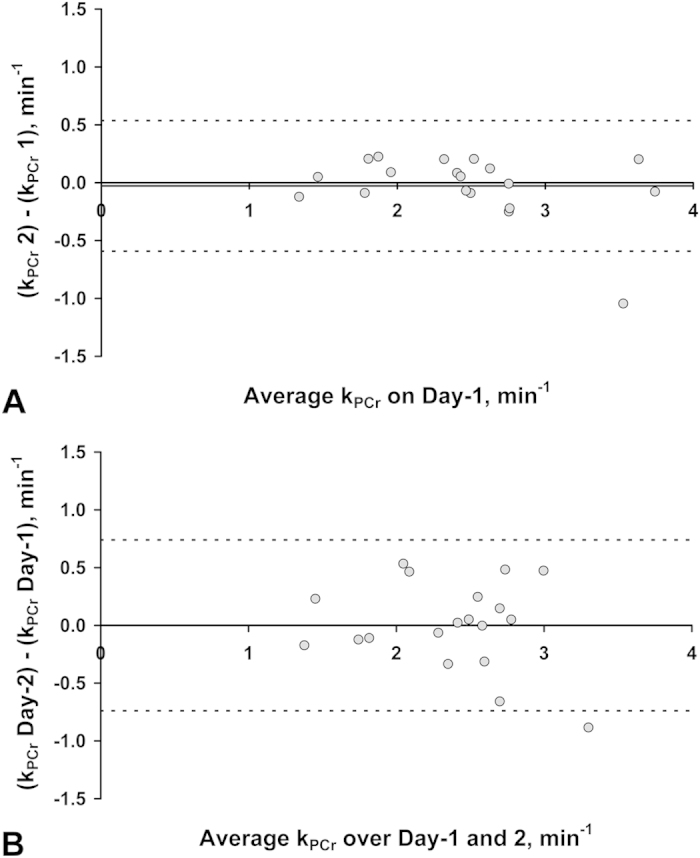
Bland-Altman plots for phosphocreatine recovery rate constant (k_PCr_) test-retest measurements in 19 healthy volunteers. Bland-Altman analysis of k_PCr_ both within (**A**), and between (**B**), scan sessions. The plots show an insignificant mean bias (solid horizontal line) of –0.03 min^−1 ^in (**A**) and 0.00 min^−1^ in (**B**), and 95% limits of agreement (dashed horizontal lines) of –0.592; 0.537 min^−1^ and –0.739; 0.740 min^−1^, in (**A**) and (**B**) respectively.

**Figure 7:**
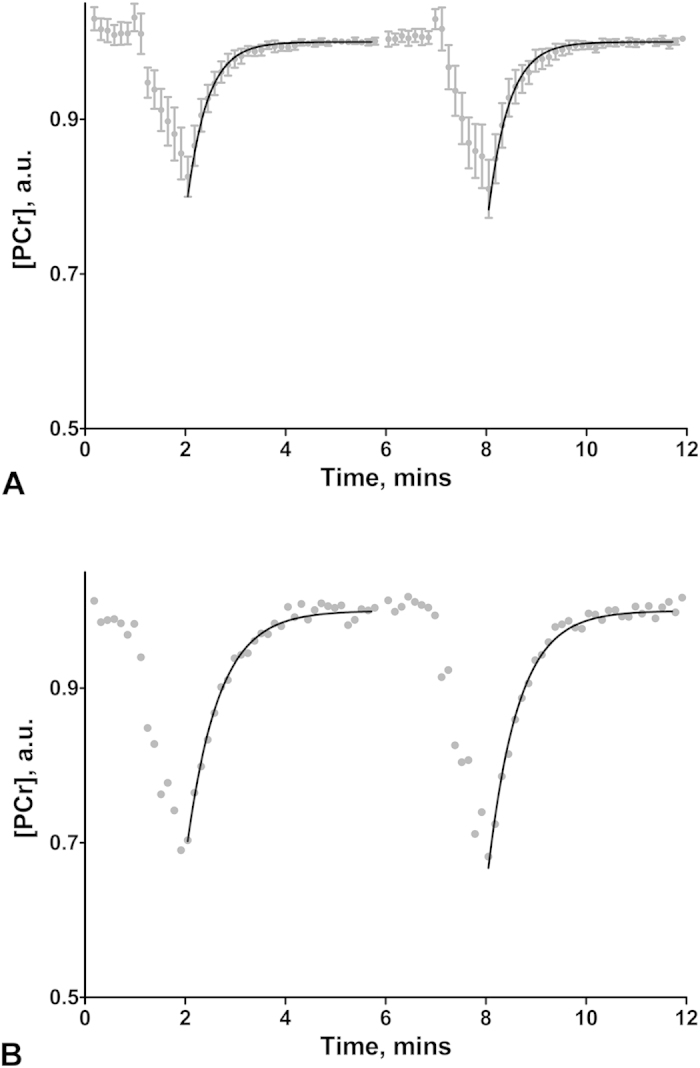
Phosphocreatine (PCr) timecourse in 19 healthy volunteers and in a lipodystrophic patient performing the optimised exercise protocol for measurement of PCr resynthesis rate post exercise. Data acquired on a Siemens MAGNETOM 3T Verio (Erlangen, Germany) in (**A**) 19 healthy volunteers and (**B**) a lipodystrophic patient performing the optimised exercise protocol of 1 min of rest, 1 min of exercise, followed by 4 minutes of rest, which is then all repeated. Grey circles represent [PCr] (mean [PCr] ± 95% confidence interval of the mean in (**A**)), determined from ^31^P-MRS spectra with a TR of 2 s, that have been quantified and then averaged to an effective TR of 8 s for clarity in this figure. In (**A**) participants [PCr] was normalised to their fully recovered post exercise PCr values (post exercise 8 s averaged time points 155–219 s inclusive), to permit intersubject comparison. The solid line represents the mono-exponential fit of the (mean in (**A**)) recovery rate constant to the PCr resynthesis data post exercise bouts one and two, with corresponding rate constants (k_PCr_) 2.47 ± 0.17 min^−1^ and 2.44 ± 0.15 min^−1^ respectively in (**A**), and 1.58 min^−1^ and 1.66 min^−1^ respectively in the lipodystrophic patient (**B**).

We have implemented this exercise design in two MR scanners with differing bore dimensions (Siemens MAGNETOM 3 T Trio (Fig. 4) and 3 T Verio (Figs 5, 6, 7), Erlangen, Germany), demonstrating the ease of translating this method across MR scanner models.

### Exercise MRS protocol

Figure 4A illustrates well controlled PCr depletion during this exercise, and demonstrates the exponential PCr decline expected during relatively low power exercise (as emphasised by dashed lines showing very little change from 1 to 2 minutes of exercise). Figure 4B shows the corresponding intracellular pH timecourse which reveals an insignificant pH change relative to basal after 1 minute, but a noticeable pH decrease after 2 minutes of exercise. Thus an exercise duration of 1 minute was chosen to minimise participant effort (which also incidentally aids compliance) and reduce the complicating effects of pH change on the interpretation of PCr recovery kinetics^5^. It is not necessary to establish a stable PCr before measurement of PCr recovery as the interpretation of k_PCr_ is independent of this^5^. In order to acquire two PCr recovery measurements which can then be averaged to improve precision, or allow two attempts at the measurement in patient groups who may be less tolerant or compliant of the exercise, the optimised protocol to measure *in vivo* mitochondrial function consisted of two 1 minute exercise durations (total scan 12 mins consisting of 1 min rest, 1 min exercise and 4 mins recovery, which is then all repeated).

### Ankle weight calculation

One scan was lost due to hardware failure, leaving data from 23 individuals who underwent the optimised exercise protocol on Day-1 using an ankle weight calculated from their MVC (Methods section). Figure 5 illustrates the consistency of the percentage PCr depletion generated over the range of MVC’s measured (79–233 Nm). Mean percentage PCr depletion relative to basal was 20.8 ± 1.3%. Average PCr depletion significantly correlated with MVC (Pearson’s r = 0.482, p = 0.02), though not when the possible outlier at high MVC was eliminated (p = 0.16).

### Reproducibility of the PCr recovery constant

Three individuals did not undertake the ^31^P measurement on Day-2 and two ^31^P scans were lost due to hardware failure (one each day). 92% of all exercise bouts were participant compliant. The PCr linewidth was 6.6 ± 0.5 Hz on Day-1. Figure 6 shows Bland-Altman plots for test-retest analysis of k_PCr_ repeatability (Fig. 6A) and k_PCr_ reproducibility over the two days (Fig. 6B), in the 19 healthy individuals. There was no significant mean bias, nor proportional bias in either Fig. 6A,B. The mean CV of repeatability and reproducibility was 5.0 ± 1.0% and 8.2 ± 1.5% respectively. The mean PCr depletion of the Day-1 and Day-2 test sessions was 20.8 ± 1.0% and 19.2 ± 1.5% respectively. Figure 7A demonstrates the mean PCr timecourse from the 19 volunteers’ Day-1 visit, and illustrates the consistency in mean PCr depletion at end of exercise bouts 1 and 2 (20.0 ± 1.3) % and (21.7 ± 1.8) % respectively. Apparent slight increases in mean [PCr] at the start of exercise bout 1 and during exercise bout 2, may reflect incorrect exercise timing due to misinterpretation of the instructions in this cohort who did not have a prescan rehearsal to the timed instructions. This is not important when we only measure and fit the PCr kinetics of the resynthesis, and this method yielded highly consistent mean k_PCr_ values of 2.47 ± 0.17 min^−1^ and 2.44 ± 0.15 min^−1^ for post-exercise recovery 1 and 2 respectively.

### Patient dataset

Figure 7B illustrates the PCr timecourse in a lipodystrophic patient performing the optimised exercise protocol for measurement of *in vivo* mitochondrial function. In this patient the ankle weight was 4.4 kg, which led to 30% and 33% PCr depletion at the end of exercise bouts 1 and 2 respectively. The k_PCr_ values were 1.58 min^−1^ and 1.66 min^−1^ for recovery 1 and 2 respectively. No significant acidification occurred after either exercise, with the pH at end exercise rising from 7.01 at basal to 7.07 (both bout 1 and 2).

## Discussion

We have described a novel and remarkably simple exercise design that allows efficient measurements of *in vivo* mitochondrial function in both healthy subjects and patients. This has been facilitated by exploiting the high density of the MR-compatible substance barium sulfate to apply load direct to the ankle, obviating the need for expensive and/or complex MR-compatible resistance or pulley equipment. The method produces well-controlled PCr depletion with patient compliance and permits highly reproducible measurements of post-exercise PCr recovery kinetics both within and between scan sessions, with coefficients of variation (5 and 8% respectively) that are comparable to the lowest reported values^29^,^30^,^31^,^32^,^33^ despite the fairly low PCr depletion in our study.

All exercise systems require a method to standardise PCr depletion between individuals and here we have done this using a measure of the volunteers’ maximal voluntary contraction assessed by a dynamometer chair, although alternative methods could be used such as a fraction of lean body mass^34^. The, on average, low fractional PCr depletion, and intersubject coefficient of variation of 30%, meant that the maximum percentage PCr depletion in visit 1 was only 33% (Fig. 5), thus the exercise was of sufficiently low intensity and duration to not yield significant acidification, which is known to slow PCr recovery^5^. This is important when using the PCr rate constant as a measure of mitochondrial function^5^, and may, in part, explain our high reproducibility values. Our ability to accurately measure the PCr kinetics post exercise at small PCr depletions is significantly aided by our high quality data (small PCr linewidths and small subcutaneous fat thicknesses in these volunteers), thus a target PCr depletion >20% may be advised in other cohorts and/or with other MR surface coils or acquisition techniques, at a field strength of 3 T.

One advantage of our exercise method is its easy-to-implement design that is readily translatable across sites and scanner models without mechanical alterations. The design does not require the participant to be strapped down to the scanner table and unlike most other procedures for exercising the quadriceps muscle *in situ*^7^,^18^,^20^,^35^ it permits the volunteer to be in the supine position which is important for patient comfort; this, together with the short exercise duration, ensures the exercise is easily tolerated. The dynamic nature of the exercise makes it suitable for patients with cardiovascular disease where forceful isometric exercise is not always recommended^36^,^37^, and the set ankle weight circumvents the need to adjust exercise intensity by interpreting force feedback which can be challenging and particularly unsuitable for those with reduced mental capacity. The apparatus’ high portability permits straightforward pre-scanning rehearsal, which could make it effective for familiarisation in cohorts with, for example, learning difficulties. These factors adapt to the needs of the participant thereby aiding compliance and making this approach particularly well suited to clinical applications.

The cost, complexity and physical demands of specialised MR-compatible ergometers appears to be limiting the wider application of ^31^P-MRS in clinical research. While detailed studies of muscle response to exercise, particularly at high intensities, requires sophisticated equipment, the assessment of PCr recovery kinetics is much less demanding and only requires a simple method to deplete the PCr pool. Our exercise design is superior to previously reported simple exercise designs^35^ in that it easily permits an individual-specific workload by altering the ankle weight, does not place participants in an uncomfortable prone position, provides a more uniform workload over displacement, and is even easier to implement. The extremely simple method we have described here provides an inexpensive, patient-focused exercise design that is highly effective and suitable for frail participants or those with reduced mental capacity, offering the opportunity to implement measurements of muscle mitochondrial function more widely in a variety of clinical trial contexts.

## Additional Information

**How to cite this article**: Sleigh, A. *et al*. Simple and effective exercise design for assessing *in vivo* mitochondrial function in clinical applications using ^31^P magnetic resonance spectroscopy. *Sci. Rep*. **6**, 19057; doi: 10.1038/srep19057 (2016).
